# Altering the localization and toxicity of arsenic in rice grain

**DOI:** 10.1038/s41598-022-09236-3

**Published:** 2022-03-25

**Authors:** Matt A. Limmer, Angelia L. Seyfferth

**Affiliations:** Department of Plant and Soil Sciences, University of Delaware, Newark, DE 19716 USA

**Keywords:** Plant sciences, Biogeochemistry

## Abstract

Previous work has shown that inorganic As localizes in rice bran whereas DMA localizes in the endosperm, but less is known about co-localization of As and S species and how they are affected by growing conditions. We used high-resolution synchrotron X-ray fluorescence imaging to image As and S species in rice grain from plants grown to maturity in soil (field and pot) and hydroponically (DMA or arsenite dosed) at field-relevant As concentrations. In hydroponics, arsenite was localized in the ovular vascular trace (OVT) and the bran while DMA permeated the endosperm and was absent from the OVT in all grains analyzed, and As species had no affect on S species. In pot studies, soil amended with Si-rich rice husk with higher DMA shifted grain As into the endosperm for both japonica and indica ecotypes. In field-grown rice from low-As soil, As localized in the OVT as arsenite glutathione, arsenite, and DMA. Results support a circumferential model of grain filling for arsenite and DMA and show Si-rich soil amendments alter grain As localization, potentially lessening risk to rice consumers.

## Introduction

Arsenic is a global contaminant of rice (*Oryza sativa* L.) due to the presence of As in most soils, the flooded soil conditions under which rice is grown, and the propensity of rice to efficiently accumulate silicon—a chemical analog of arsenite^1^. However, the presence of several different As species present in porewater and in plant tissues complicates rice accumulation of As. Arsenic in its inorganic forms (trivalent arsenite and pentavalent arsenate) are group 1 carcinogens^2^, and arsenite is routinely found in rice paddy porewater, rice plants, and rice grain^1^. Inorganic As can be methylated by soil microbes^3,4^, resulting in the pentavalent species monomethylarsonic acid (MMA), dimethylarsinic acid (DMA), trimethylarsine oxide (TMAO), and tetramethylarsonium. While all of these methylated forms have been reported in rice grain, DMA is the most common^5–7^. DMA can cause straighthead disorder in rice^8^, but this yield impact can be reversed with increasing Si^9^. These methylated forms are less acutely toxic to humans than inorganic As, but the carcinogenicity of the methylated species requires additional study, as DMA and MMA are possible carcinogens^2,10^. Understanding how different As species accumulate and localize in rice grain has implications for how rice and rice products affect human health and influences mitigation strategies.

The relative concentrations of organic As and inorganic As in rice grain can be affected by a number of factors, with consequential risks to human health as countries increasingly focus regulations on grain inorganic As rather than grain total As. One clear factor that affects the percentage of inorganic As in rice grain is the concentration of total As in the soil, with increasing soil As increasing the fraction of organic As in rice grain^11^. Increasing soil As is thought to increase microbial methylation, resulting in increased availability of methylated species to rice plants, which are readily translocated to shoots and grain^12^. Similarly, market basket surveys have shown that DMA concentrations in rice grain are correlated to grain total As, indicating that as grain total As increases, most of the increase in total As results from the increase in grain DMA^5^. Geography has also been reported to affect grain As speciation, with grain from the mid-south United States previously reported to have higher fractions of DMA^5^, although substantial variation exists within states^13^, casting doubt on the validity of broad geographic generalizations. Another factor that can affect As speciation in rice grain is the application of Si to soil, a crucial nutrient for rice^14^ that can have contrasting effects on plant uptake of As species^15^. Because arsenite enters rice roots through Si transporters^16^, increased plant-available Si decreases inorganic As in rice grain^11,17–21^. However, increased plant-available Si can increase grain organic As, likely through Si-induced desorption of arsenite in the porewater^22^ leading to increased microbial methylation^11,23^. Despite a growing body of literature on the factors that affect grain As concentration and speciation, few studies investigate factors that affect the localization of As in grain.

The localization of As species in rice grain remains an important area of study because the extent of As permeation into the endosperm affects the efficacy of As removal by polishing, the de facto treatment process for decreasing human exposure to As via rice. Early work identified strong localization of As in the edge of the grain^24,25^ or in the bran^26,27^. Additional work showed that As was most concentrated in the ovular vascular trace (OVT)^28–31^, the primary vascular bundle filling the grain^32^. Many of these studies imaged relatively thick grain sections, which hinders detailed interpretation due to parallax and attenuation of low Z fluorescent X-rays with depth. Arsenic can also be present in the subaleurone layer^33^, which is less likely to be removed by polishing. Studies with excised grain^34^ and hydroponic rice^35^ showed DMA readily dispersed into the endosperm while arsenite was retained in the OVT^34,36^. However, similar work with flag leaf arsenate exposure showed a concentration-dependent response, with arsenate at low concentrations retained in the OVT, while at higher concentrations arsenate permeated the grain^37^. This highlights the importance of studying elemental distribution in rice with field-relevant concentrations, in contrast to highly contaminated rice which is more often studied due to the ease of analytical detection.

Arsenic speciation in rice grain is complicated by the interactions of As with sulfur. Sulfur, a yield-limiting nutrient^38,39^, is present in rice grain at high concentrations (~ 0.1%) within S-rich amino acids methionine and cysteine and derivatives primarily as glutathione^40^. Arsenite has a high affinity for thiol groups and forms stable complexes *in planta* with glutathione^41^. However, these complexes are not stable during most routine extractions and/or analytical techniques and instead are typically measured as free arsenite^42^. However, arsenite-glutathione complexes (As(III)(GS)_3_) can disassociate during human digestion, so mischaracterizing arsenite-glutathione as arsenite may be of little consequence to human health^28^. Thiolated As may also be present in rice grain, but common extraction methods can result in the loss of the sulfur group, leading to mischaracterization of the As species^43–45^. Sequestration of arsenite glutathione in plants has led some to propose adding S to rice soils to decrease As accumulation in the grain^46–48^; however, widespread efficacy of these treatments remains unclear as well as the significance of thiolated As species in rice grain. More broadly, how As affects grain S homeostasis is unclear.

The goal of this work was to examine the localization of As species in rice grain from plants grown to maturity under a variety of realistic environmental conditions including those grown with Si-rich soil amendments. We used synchrotron radiation microprobe X-ray fluorescence (SR-μXRF) imaging to examine grain from plants grown to grain maturity either hydroponically with realistic arsenite or DMA exposure or in soil with varying, field-relevant levels of As. We hypothesized that arsenite would be concentrated in the bran and OVT, independent of planting media tested and cultivar. In contrast, we hypothesized DMA would permeate farther into the endosperm. We also hypothesized that arsenite exposure would disrupt grain S homeostasis, altering sulfur speciation and localization in grain due to complexation with glutathione. Finally, we hypothesized that soil treatments with increased Si would result in increased permeation of As into the grain. Synthesis of these results and literature support the conceptual model of circumferential loading of DMA and arsenite into the grain, resulting in a distribution of grain As that primarily depends on the concentration of individual As species. Moreover, our data suggest that soil incorporation of Si-rich rice husk may be a globally relevant way to produce nutrient-rich and low-iAs brown rice or rice bran and related products, which has an advantage over the existing strategy to meet grain As health standards by dilution of high As rice with low As rice.

## Results

### DMA and arsenite are localized and concentrated differently in rice grain with similar patterns of S

**Figure 1:**
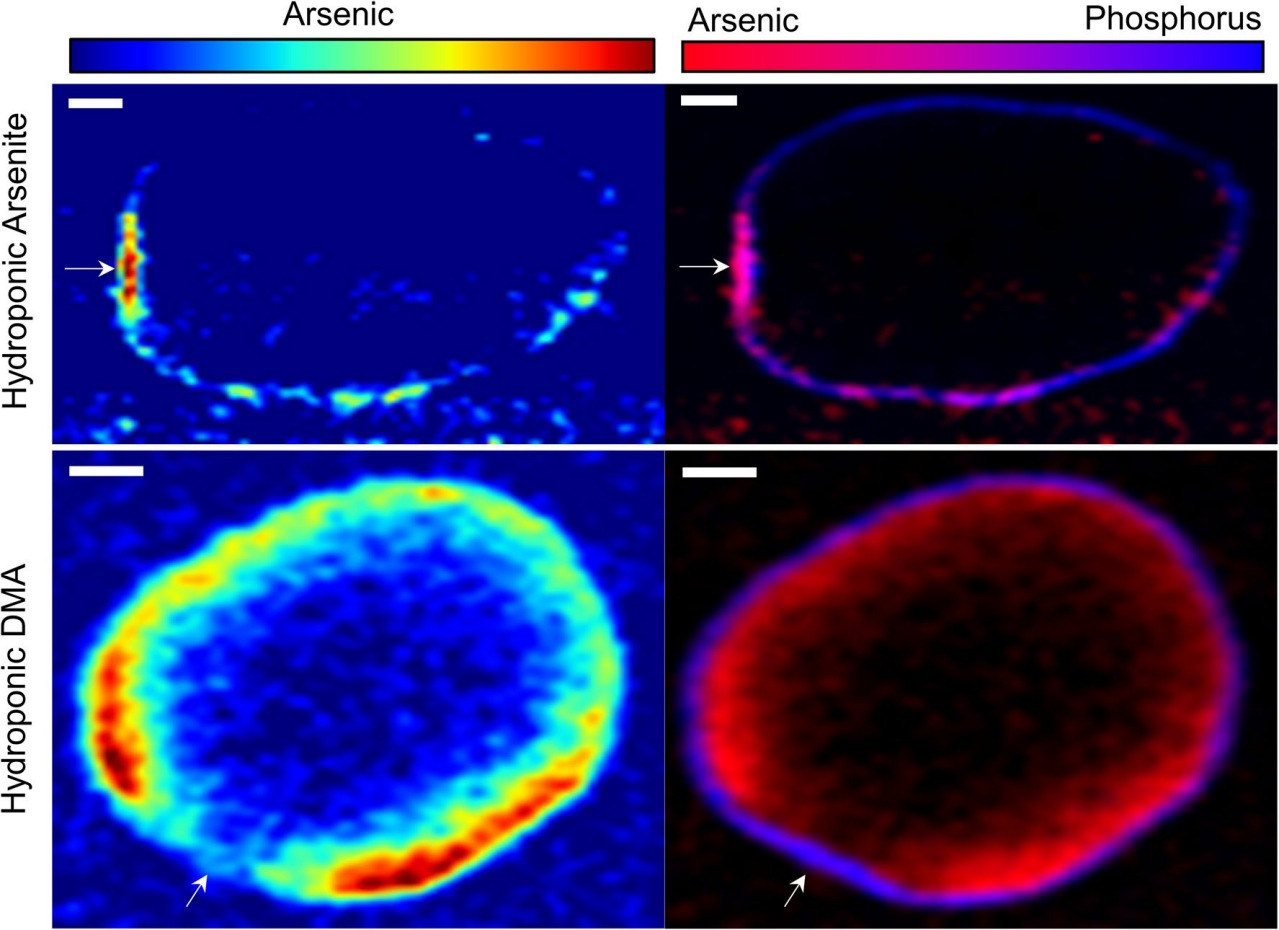
Distribution of As (left) and colocalization (right) of As (red) and P (blue) in rice grains grown hydroponically receiving either arsenite (8 uM) or DMA (5 uM). In the grain receiving arsenite, As is mainly located in the bran and the OVT (arrow) as shown by purple hues in the bicolor plots. In the DMA treatment, As permeates into the endosperm and is notably low near the OVT (arrow). Scale bar is 300 μm.

To isolate the impacts of As speciation on localization in rice, we grew rice to seed maturity with field-relevant levels of DMA (5 μM) or arsenite (1, 4, or 8 μM) in nutrient solutions that approach soil solution conditions (Table S1). The chemical form of As (arsenite or DMA) in hydroponic culture resulted in different As concentrations and patterns of localization in the unpolished grain (Fig. 1). In the arsenite treatment, As reached levels of only 0.16 ± 0.03 mg kg^−1^ As for all arsenite treatments combined and speciation analysis using dilute HNO_3_ extraction and HPLC-ICP-MS confirmed that 97% of the total As was present as inorganic As. This As was strongly localized to the OVT and bran; any As in the endosperm was at concentrations similar to the background (i.e., below the detection limit). Even though, unlike DMA treatments, the arsenite was only provided during reproduction, arsenite treatments exhibited As phytotoxicity with leaf chlorosis and lower grain yield for higher solution As concentrations: the 1 μM As treatment yielded 10.7 g pot^−1^ and the 8 μM As treatment yielded 5.4 g pot^−1^ on average. In contrast to arsenite treatments, the concentrations and localization of grain As in the DMA treatment differed substantially, with an order of magnitude higher levels of grain As of 1.24 ± 0.14 mg kg^−1^ of As (± standard deviation, n = 3). Speciation analysis confirmed that 99% of the As in the grain was DMA, and this As was displaced farther into the endosperm (Fig. 1). However, some As was still present in the bran, as evidenced by colocalization with P (purple in Fig. 1). The correlation between P and As was much weaker for grain from DMA treatment as compared to arsenite treatment (Fig. S1a). Very little As reached the center of the endosperm, but rather was concentrated immediately interior to the bran. More As was in the half of the grain closer to the OVT (i.e., ventral), but the OVT had relatively low concentrations of As. Analysis of additional grains from these experiments showed similar distribution patterns (Figs. S2 and S3).

**Figure 2:**
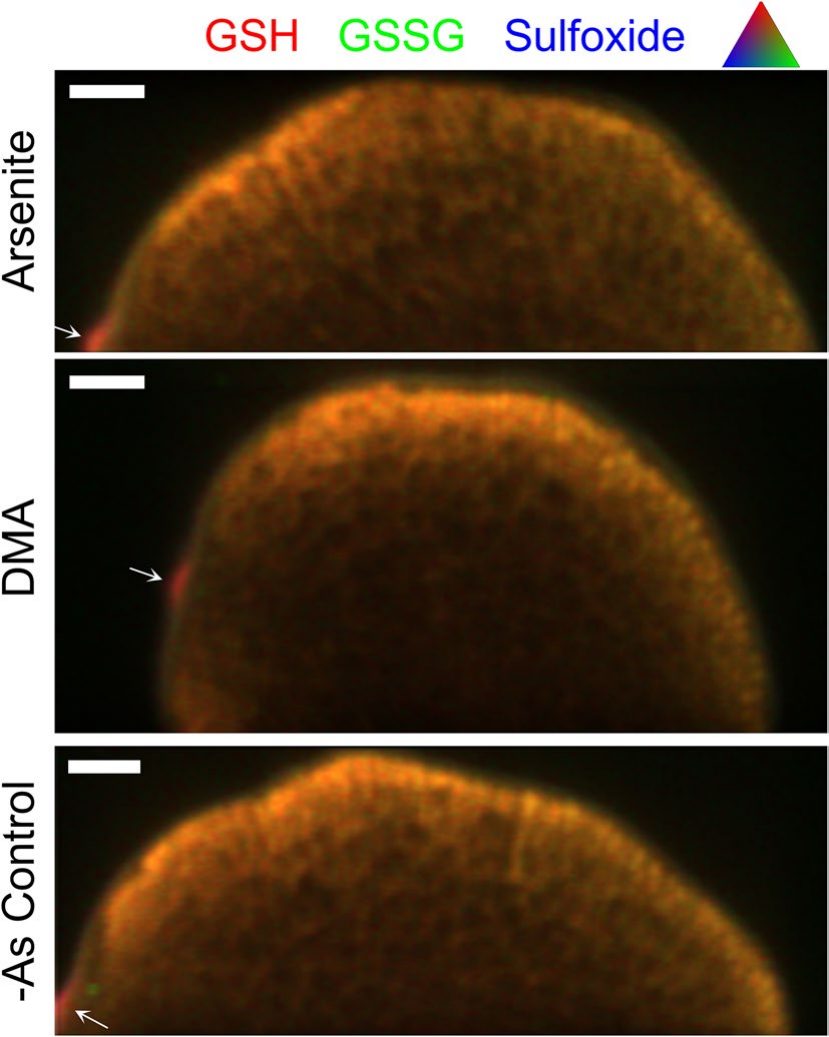
Distribution of S species in hydroponic rice receiving either arsenite (1 uM), DMA (5 uM), or no As. Arrows show the location of the OVT. All S species are scaled to the same maximum value to illustrate relative concentrations. Scale bars are 200 μm.

Sulfur speciation and distribution was similar between the three hydroponic As treatments (DMA, arsenite, and -As). Total sulfur (1.3–1.5 g kg^−1^) was highest near the bran but permeated into the endosperm with decreasing intensity (Fig. 2). Using S XANES, the reduced S compounds cysteine, methionine, and GSH had similar spectra and are collectively referred to as GSH here. Similarly, cystine and GSSG had similar spectra and are referred to as GSSG here. The speciation of grain S for sparse energy excitation X-ray absorption spectroscopy (SEE-XAS) was constrained by μXANES at select points on the grain and linear combination fitting against several biological forms of S (Fig. S4). In the bran of both As treatments, μXANES fit 69 ± 6% GSH, 17 ± 3% GSSG, 7 ± 2% DMSO, and 6 ± 6% APS (average ± standard deviation, n = 6, Fig. S5). Two sulfoxide compounds were used as standards, with DMSO fluorescence shifted to slightly higher energy than MetO (Fig. S4). DMSO resulted in slight improvements in the linear combination fitting and was used as the preferred sulfoxide standard for these samples. In the grain SEE-XAS maps, S was primarily GSH and GSSG, although sulfoxides and APS were also detected in lesser amounts (Fig. 2, Figs. S6, S7, and S8). In tricolor plots of S species, the ratio of sulfur species remained relatively constant throughout most of the grain, except for the OVT (Fig. 2). Much of the grain was dominated by GSH, as shown by the red/orange hue in the tricolor plots.

### Growing conditions alter the speciation and localization of As in rice

**Figure 3:**
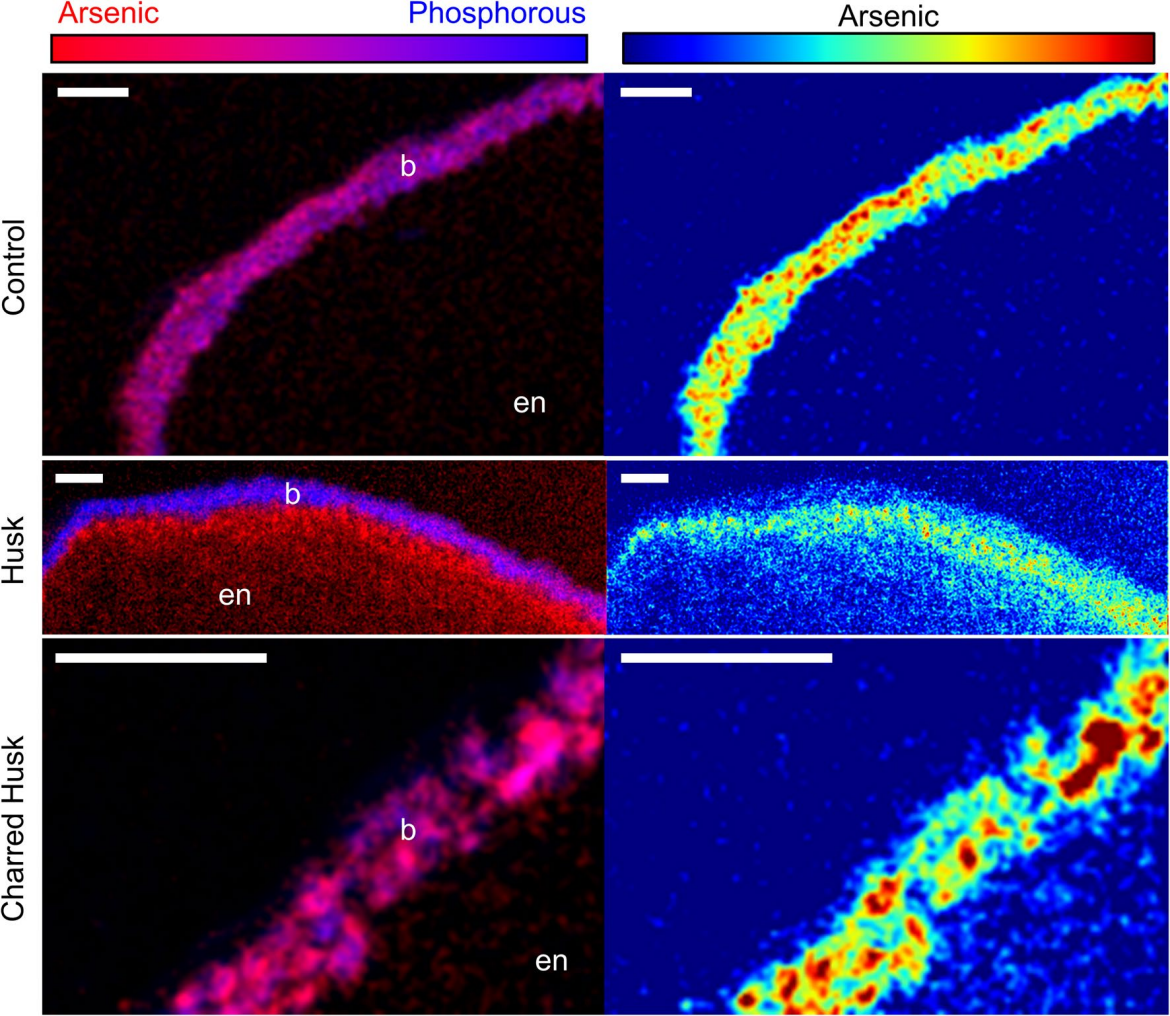
Effect of Si-rich soil amendments on As localization in M206 rice grains. Bicolor plots show distribution of As (red) and P (blue) at the edge of a longitudinal thin section. For As plots, warmer colors correlate with higher concentrations of As. Grain from each plant was 53%, 85%, and 55% organic As for control, husk, and charred husk treatments, respectively. Scale bar is 100 μm. b, bran; en, endosperm.

**Figure 4:**
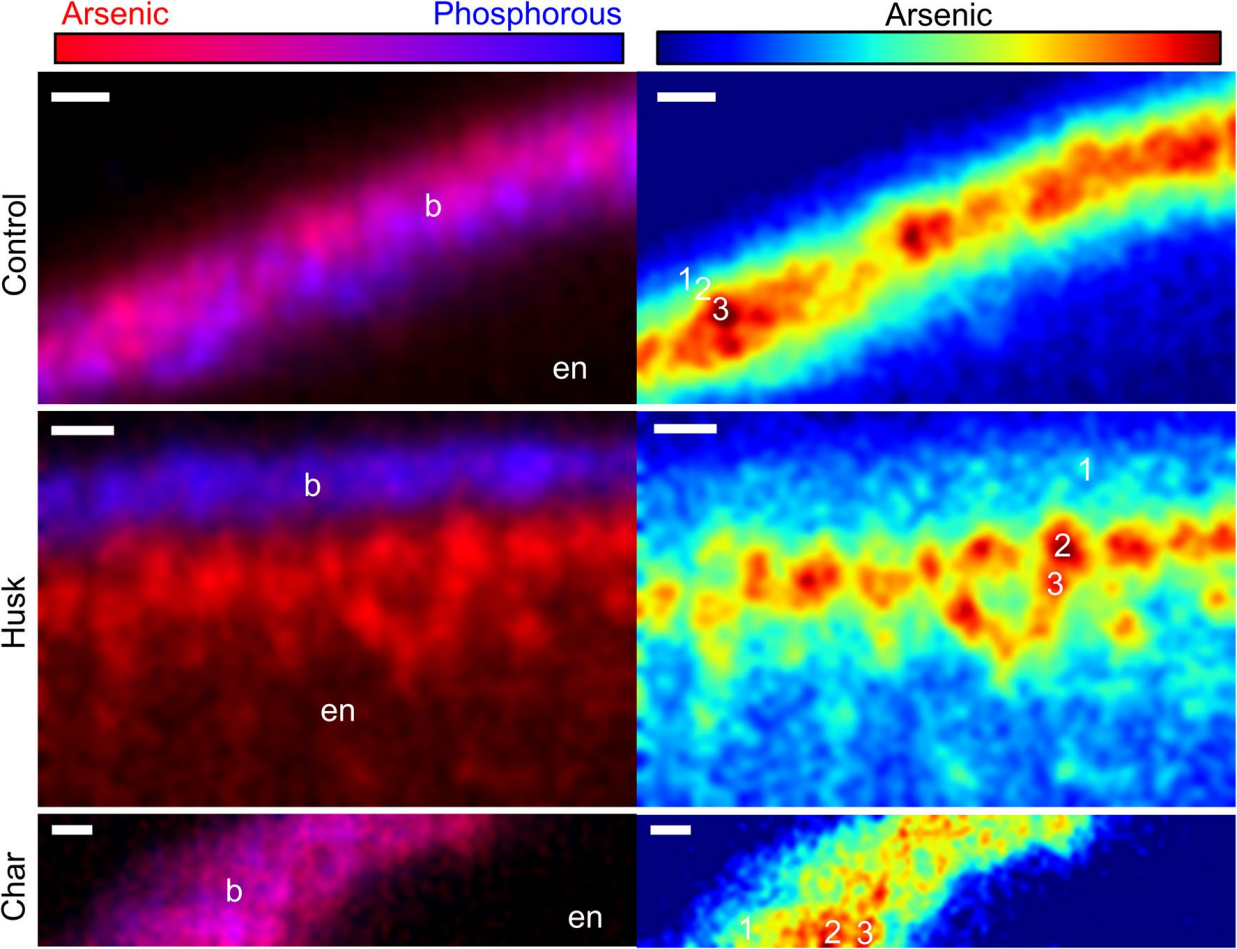
Effect of Si-rich soil amendments on As localization in IR66 rice grains. Bicolor plots show distribution of As (red) and P (blue) at the edge of a longitudinal thin section. For As plots, warmer colors correlate with higher concentrations of As. Grain from each plant was 44%, 83%, and 39% organic As for control, husk, and charred husk (‘Char’) treatments, respectively. Numbered locations indicate μXANES scans and linear combination fit results are shown in Table S3. Scale bar is 20 μm. b, bran; en, endosperm.

The presence of Si-rich amendments in soil-grown rice affected the As speciation and localization in the grain. We examined localization of grain As from both indica and japonica rice that had been grown in husk- or charred husk-amended soil having 17 mg kg^−1^ As in a pot study^21^. Husk amendment significantly decreased grain inorganic As by 40–50% but either did not change or increased DMA concentrations, resulting in rice grain As with 79 ± 6% DMA for cultivar M206 (japonica) and 71 ± 10% for IR66 (indica) (average ± standard deviation, n = 3) without affecting yield. The charred husk treatment tended to decrease both inorganic As and DMA, resulting in percentages of grain DMA of 48 ± 17% for M206 and 56 ± 16% for IR66; while the concentrations were lower, the percentages of DMA were similar to the unamended control^21^. The changes in grain As speciation in the pot study corresponded to changes in As localization, with DMA-rich grains from husk amendments having As less localized to the bran (Figs. 3, 4). The high-resolution SR-μXRF images revealed that most of the As was collocated with the phosphorous present in the bran in both the control and charred husk treatment (Fig. S1c), whereas As was more diffuse and permeated farther into the endosperm in the husk treatment, and this effect was observed both in the japonica M206 (Fig. 3) and in the indica IR66 (Fig. 4) ecotypes. Linear combination fitting of μXANES data from IR66 found most As in the bran was either arsenite or arsenite glutathione in the control and charred husk treatment (88% on average), but 36% of the As was present as DMA in the husk treatment (Table S3). These data illustrate that rice grown in husk-amended soils shifts As speciation toward DMA, which is localized away from bran and farther into the endosperm.

**Figure 5:**
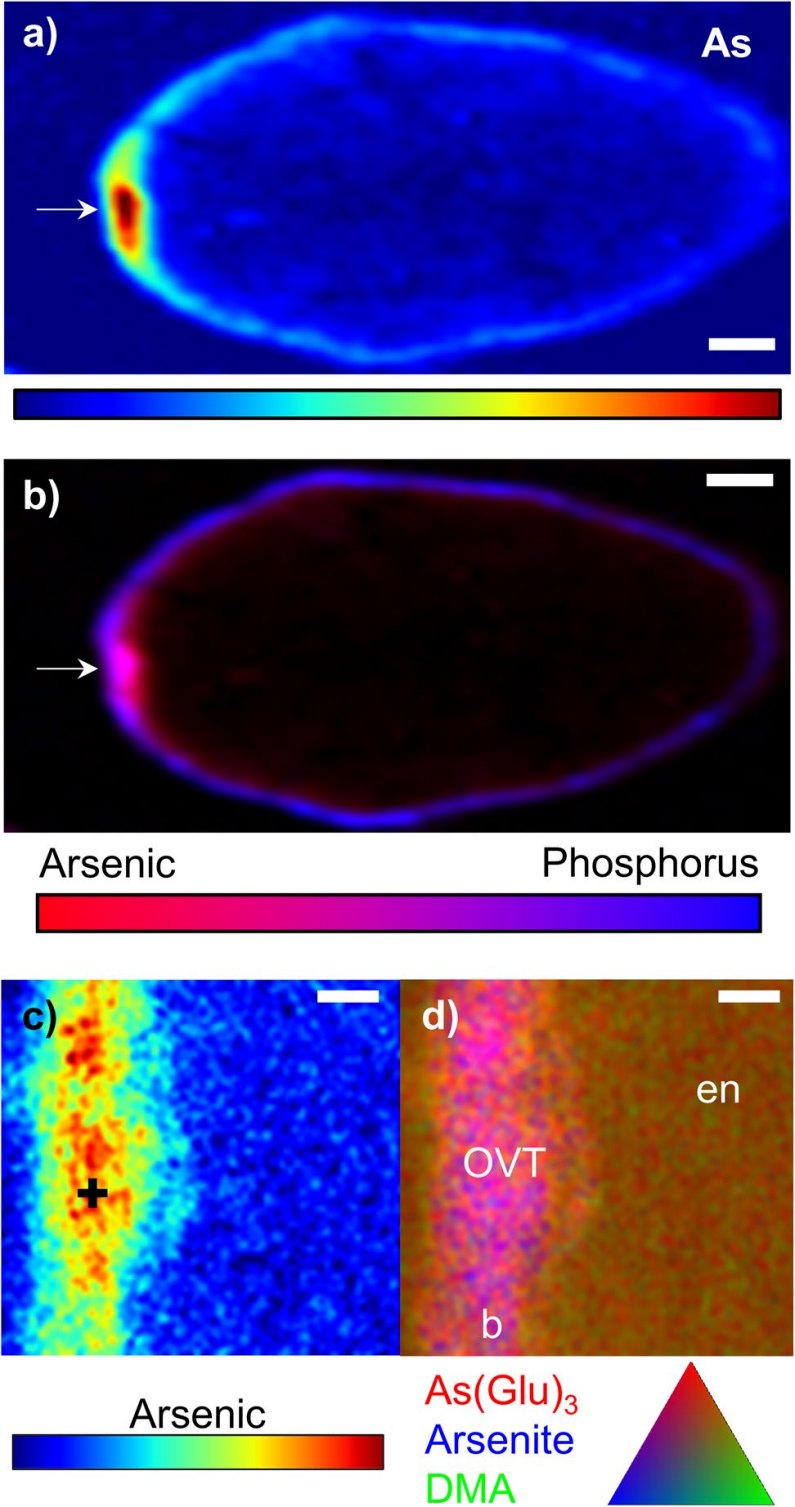
Distribution of total and speciated As in CLXL745 hybrid rice grown under field conditions. (**a**) Distribution of As in grain transverse-section. Scale bar is 300 μm. (**b**) Bicolor plot of As (red) and P (blue), where the arrow denotes the OVT. Scale bar is 300 μm. (**c**) High-resolution map of As from OVT area in b). Warmer colors are proportional to higher concentrations of As. Black + denotes location where μXANES scans were taken. Scale bar is 50 μm. (**d**) High-resolution As speciation map around the OVT, where all As species are scaled to the same maximum value. Scale bar is 50 μm. en, endodermis; b, bran.

We also examined field-grown hybrid rice that had been grown in delayed flood from Arkansas, which had a relatively even mixture of inorganic and organic As. The plot averaged 0.76 mg kg^−1^ As in grain, of which 43% was organic As in the form of DMA. Most of the As was localized in the OVT, although the bran was also enriched in As (Fig. 5a and Fig. S1b). Most of the co-localized As and P was present in the OVT (Fig. 5b), and As permeated farther into the endosperm than P. A high resolution map of As in the OVT (Fig. 5c) and an As SEE-XAS speciation map (Fig. 5d) showed that As in the OVT was predominately arsenite and arsenite glutathione. DMA was much more diffuse and was present in the endosperm. Linear combination fitting of 9 replicate μXANES spectra taken in the OVT found 30 ± 2.6% arsenite glutathione, 60 ± 3.8% arsenite, and 10 ± 4.6% DMA (average ± standard error from LCF fit, r-factor: 0.024, Fig. S9).

## Discussion

### Localization of As species in grain

While our data support the work of others that show localization of As in the bran and OVT, we found that As localization is strongly affected by the As species present, which can be affected by growing conditions. In agreement with our hypothesis, hydroponic rice exposed to arsenite retained As in the bran, primarily in the OVT regardless of arsenite treatment from 1–8 uM (Fig. 1, Figs. S2 and S3), and the As was collocated with P (Fig. 1 and Fig. S1). Phosphorus in rice grain is in the form of phytic acid and is located in the aleurone layer, allowing grain P to be used in bran delineation^49,50^. Some As was also present immediately interior to the P, although these signals approached background levels. Using a cut panicle exposed to arsenite, arsenic has been shown to accumulate in the subaleurone layer^33^, although grain in that experiment were pulsed with 133 μM arsenite, which is much higher than found in rice paddy porewaters or plants. In similar types of experiments using excised grain or through cut flag leaves fed with inorganic As, inorganic As has been reported to primarily accumulate in the OVT^34,36,37^. Arsenite has also been shown to be located in the OVT of hydroponic rice grown with arsenate^35^, although SR-μXRF analysis of thick sections complicates interpretation at fine scales. Here we show that under more field-relevant As concentrations using intact plants, hydroponic rice exposed to arsenite during reproductive growth strongly localizes As in the OVT (Fig. 1, Figs. S2, and S3). We observed a similar localization of As in field-grown rice that had a higher proportion of arsenite than DMA, where a majority of the As was localized to the OVT and most of this As was arsenite or arsenite glutathione (Fig. 5 and Fig. S9).

In contrast to the strong localization of arsenite in the OVT and bran, DMA localizes in the endosperm. In agreement with our hypothesis, hydroponic rice grown with DMA showed substantial As permeation into the endosperm (Fig. 1). Arsenic was most concentrated adjacent to the bran layer as delineated by P, although As was notably absent from the OVT. Previous work with excised grain pulsed with DMA showed that As dispersed into the endosperm, although the bran retained some DMA except near the OVT^34^. In a hydroponic study, DMA was located throughout the grain, but the thickness of the sections (1/2 or 1/3 of grain) complicated a more detailed description^35^. Taken together, these data support a more diffuse DMA localization in the endosperm as compared to arsenite. These results also show that the distribution of As species were not strongly influenced by the As exposure method—through roots (via soil or hydroponic solution in this work), cut flag leaves^36,37^, or cut panicles^34^—although concentration-dependent effects are more difficult to assess using different dosing methods.

### Effect of Si-rich amendments on As localization

In support of our hypothesis, Si-rich husk-treatments strongly affected grain As localization in both ecotypes, and the change in localization of As was consistent with changes in grain As speciation caused by Si. Increased porewater Si from husk amendment can decrease rice uptake of arsenite, but Si can also increase accumulation of DMA in rice grain^11,16^, despite DMA entering rice roots through the silicon channel Lsi1 and hydroponic work demonstrating Si can decrease grain concentrations of DMA^9,51^. Exogenous Si leads to increased microbial methylation of As likely due to Si-induced desorption of inorganic As and consequent methylation^23^. This results in more DMA available for plant-uptake upon Si-rich husk amendment. The grains imaged here were from pot studies where Si was applied to soil with elevated As (17 mg kg^−1^) using either rice husk, which led to the highest porewater Si concentrations, or charred rice husk, which led to lower porewater Si concentrations due to crystallization of Si upon charring but still higher than unamended control^21,52,53^. The husk treatment increased the percentage of methylated As in the grain without substantially changing the total grain As or affecting yield^21^. In contrast, the charred husk treatment resulted in a similar fraction of DMA in the grain to the untreated control, providing a sample set to test the effect of Si on grain As localization in two different ecotypes of rice. The distribution of As in the DMA-rich grain (i.e., husk treatment) strongly shifted to the interior of the grain for both ecotypes (Figs. 3, 4). In the husk treatment, grain As was more diffuse, similar to the distribution observed for DMA-dosed plants grown hydroponically (Fig. 1). In contrast, the control and charred husk treatments showed nearly complete colocalization of P and As in the bran layer, consistent with the higher fraction of inorganic As in the grain. Because this change in As distribution was consistent across two different ecotypes of rice, this demonstrates an edaphic control on this process. These results suggest that soil amendments of Si-rich rice husk can decrease inorganic concentrations in brown rice and produce rice bran products with minimal As.

### Interactions between As and S

The affinity of arsenite for thiol groups results in stable arsenite glutathione/phytochelatin complexes formed *in planta*, here referred to as arsenite glutathione^41,42^*,* which led us to probe the interactions between As and S in rice grain. Notably, dilute acid extractions commonly used to extract As species result in disassociation of the complex, so the presence of these complexes is more easily measured using XANES. Reports of As speciation using μXANES in rice grain vary widely, with some reporting relatively high proportions of arsenite glutathione (~ 50%)^28^ while others reporting no evidence of arsenite glutathione in rice grain^26,27^. Work with bulk XANES found that a majority of inorganic As was present as arsenite glutathione, and low concentrations of arsenate or arsenite were occasionally observed^54^. In this work, As spectroscopy was most successful in the grain OVT and bran, where As was strongly concentrated (Figs. 4, 5). SEE-XAS of the OVT of field-grown rice revealed the high concentration area of As was a mixture of arsenite and arsenite glutathione, while DMA was more diffuse (Fig. 5d). Linear combination fitting of μXANES in the OVT confirmed the results of the SEE-XAS, with the OVT dominated by arsenite and arsenite glutathione with lesser amounts of DMA (Fig. S9). Similar results were found in the pot study, with the bran predominately arsenite glutathione and arsenite (Table S3). We also observed some differences in the fraction of arsenite associated with glutathione, although this effect requires additional study. Despite the ability of XANES to distinguish arsenite glutathione from arsenite, XANES has limited ability to distinguish some As compounds, including overlapping white lines between some methylated As species and thiolated As species^55,56^, which becomes problematic when multiple spectroscopically-similar species are present at low concentrations^34^. Thus, detection of thiolated As species in rice grain by XANES will likely be difficult under field-relevant conditions. While XANES remains appropriate for measurement of some major As species in rice grain, chemical extraction and chromatographic separation are more appropriate for quantifying most As species at low concentrations^45^.

The interaction between arsenite and glutathione in rice led us to hypothesize that hydroponic plants exposed to arsenite would affect the distribution of S species in the grain compared to plants receiving DMA or -As. However, the relative proportions of S species and their distribution remained similar in the grain between the treatments (Fig. 2). Sulfur has previously been shown to permeate into the grain^28^, as observed here. We also observed that the speciation of S remained similar in the bran and further into the grain. Additionally, S speciation was dominated by reduced amino acid S, denoted as GSH because reduced S in rice grain has previously been shown to consist mostly of GSH with lesser amounts of cysteine and methionine^57^. Glutathione is ubiquitous in rice, as S is transported in rice phloem as glutathione^58^ where it is stored in the grain in amino acids^40^. We also observed lower amounts of oxidized S amino acids and sulfate stored in the form of APS in the grain. Despite the arsenite-induced oxidative stress which led to decreased grain production, the grain was able to maintain speciation of S similar to a DMA-treated and -As control plant.

### Grain loading of As species

**Figure 6:**
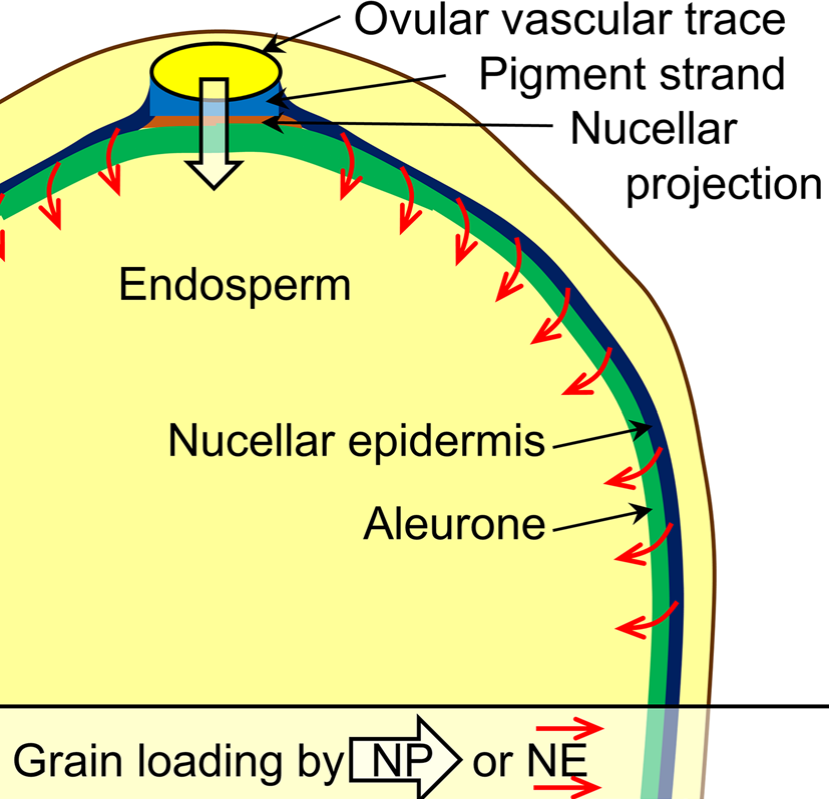
Pathways of nutrient loading into a developing rice grain. The grain is loaded from the ovular vascular trace, with nutrients following two potential pathways into the grain. Initially, grain loading may be dominated by crossing the pigment strand and nucellar projection to enter the filial tissues of the aleurone and endosperm. Later in grain filling the circumferential pathway dominates, with nutrients traveling through the nucellar epidermis around the grain to enter the filial tissues of the aleurone and endosperm. Both DMA and arsenite primarily follow circumferential loading. Image created using Adobe Illustrator.

The predominance of As in rice bran suggests that most of the As follows the circumferential grain loading pathway. All elements enter through the phloem and xylem of the OVT, then follow one of two pathways farther into the grain (Fig. 6). One pathway is via circumferential loading, where solutes flow through maternal tissues from the OVT into the adjacent pigment strand and spread around the grain through the nucellar epidermis, followed by centripetal transport into the aleurone and the endosperm^59–61^. Sucrose is a molecule that has been shown to enter via another pathway by directly entering the grain through the nucellar projection^62,63^. This direct pathway into the grain is thought to dominate during early grain filling, with circumferential transport predominating later in grain filling^32^. Arsenite here and in other studies is strongly localized in the bran aleurone layers, which are thickest near the OVT^64^. The stability of arsenite glutathione complexes^42^, their storage in vacuoles^41^, and their prevalence in rice grain (Fig. 5) suggest that as inorganic As enters the grain, the aleurone sequesters the As with glutathione, preventing further translocation into the grain. Inorganic As has occasionally been observed farther into the grain in the subaleurone layer^33^, although the extent of inorganic As permeation is likely dose-dependent, as was observed for arsenate loading^37^. High concentrations of inorganic As may be able to overwhelm the capacity of the aleurone layer to sequester As. DMA also shows a strong circumferential loading pattern (Fig. 1), but we hypothesize that the high mobility of DMA due to limited interaction with glutathione^42^ allows DMA to more readily permeate farther into grain. Notably, DMA is relatively absent in the OVT, which may be a result of excess water leaving the endosperm out through the nucellar projection and pigment strand^60^, allowing advective flow of DMA out of the grain near the OVT. This is in agreement with the observation that DMA accumulates more quickly than inorganic As in the grain^29^, potentially allowing more advective removal of DMA, particularly near the OVT. Collectively, these As loading patterns highlight the importance of the aleurone in limiting mobility of As species in the grain, similar to other nutrients such as Zn. Thus, caution should be exercised in efforts intended to alter elemental distribution in the aleurone and endosperm, as these may affect other elements with similar distributions.

The consistent patterns of As observed across this and other studies imply that As loading is controlled by the As species present and their concentration, which provides insight into techniques to lessen human As exposure. In cases where the grain is mostly inorganic As, polishing remains a straightforward technique to produce grain with lower As, although this leaves a high-As bran. Shifting the speciation of As in grain can be beneficial due to the lower risk posed by DMA as compared to inorganic As and the more diffuse distribution of DMA, allowing low-As bran products. Unfortunately most increases in grain DMA result from increases in plant exposure to As through As-contaminated soil or soil amendments that substantially decrease the soil redox thereby increasing As availability through reductive dissolution^11^. Silicon addition to the soil via husk amendment can increase As methylation^23^, but ideal Si-rich amendments must provide limited labile carbon to prevent undesirable decreases in soil redox potential that risk increasing total As. For these reasons, we recommend rice husk instead of rice straw^65^. Additionally, Si is able to mitigate straighthead disorder induced by high DMA^8,9,66^, thereby preventing yield losses typically associated with DMA-induced straighthead disorder. Other techniques to shift the speciation of grain As toward DMA without substantially lowering soil redox and yield should be explored.

## Methods

### Rice grain

Rice grain was obtained from plants grown to maturity in hydroponic experiments and from previously published pot and field studies under a range of As concentrations^21,67^. All rice grain samples were collected following U.S. rules and regulations. In a hydroponic study, rice (*Oryza sati*va L. cv. Lemont) was grown in a growth chamber with 28 °C days (12 h) and 26 °C nights, 70% relative humidity, and fluorescent lighting, which provided > 200 µmol m^−2^ s^−1^ at the plant base. One week after germination, seedlings were transplanted to 8L high-density polyethylene pots for the remainder of the experiment. The hydroponic nutrient solution (Table S1) contained either no As (-As control) or As as DMA (as cacodylic acid) at 5 μM or arsenite (as sodium arsenite) at 1, 4, or 8 μM. To inhibit microbial transformation of arsenite, 6 μM of chloramphenicol was added to the arsenite treatments^68^. Plants received arsenite only during the reproductive growth stage to limit arsenite phytotoxicity, whereas for DMA treatments plants received DMA throughout the life of the plant. The nutrient solution was exchanged every 3–7 days, with solution changes becoming more frequent with plant growth. Three replicate plants were grown for the DMA treatment, four replicate plants were grown for the arsenite treatment per As concentration, and two replicate plants were grown for the -As control. Rice grains were also obtained from a previously published pot study^21^, where rice was grown in similar growth chamber conditions as described above, but in soil containing 17 mg kg^−1^ As. Soil treatments included an untreated control or soil amended with rice husk or charred rice husk at 1% w/w. Two different cultivars were analyzed here: M206 (medium grain Calrose rice, *japonica*) and IR66 (long grain *indica*). Rice grains were also collected from a previously published field study in Arkansas^67^. The hybrid rice (long grain CLXL745) was from a field in rice-soybean rotation using a conventional delayed flood and the soil had 4.2 mg kg^−1^ of As. From all experiments, representative rice grains were embedded in Epotek 301-2fl epoxy and 30 μm thin sections were made by Spectrum Petrographics Inc. (Vancouver, WA), which does not affect grain As speciation^27^. Thin sections were created as longitudinal and transverse sections under low oxygen conditions and adhered to quartz slides.

### Chemical analyses

Elemental composition and arsenic speciation were measured for unpolished grain. Prior to chemical analysis grain was dehusked and finely ground. For total elemental composition, grain was digested in concentrated nitric acid using microwave digestion following published protocols^21^. Samples were analyzed using ICP-MS (Agilent 7500cx). For As speciation, grain was extracted using 2% HNO_3_ following Maher et al.^54^ and was analyzed using HPLC-ICP-MS (Agilent 7500cx) following Jackson^69^. NIST 1568B was used to ensure appropriate recovery of total and speciated As. Recovery of total As was 113% and recoveries of As species were 124% for inorganic As, 85% for DMA, and 107% for MMA. Grain As concentrations and speciation are summarized in Table S2.

### Synchrotron radiation microprobe X-ray fluorescence (SR-μXRF)

Thin sections were analyzed using SR-μXRF on three different beamlines at the Stanford Synchrotron Radiation Lightsource (SSRL). Entire longitudinal or transverse sections of grains were imaged on beamline 10–2 with an incident beam size of 50 μm and energy of 13 keV. The sample was rastered across the beam at step sizes of 25 μm with a dwell time of 300 ms pixel^−1^. Areas of interest were additionally imaged at beamline 2–3 with an incident beam size of 2 μm and energy of 12 keV. The sample was rastered across the beam at step sizes of 2 μm with a dwell time of 500 ms pixel^−1^. Samples were also scanned at multiple energies (11,869, 11,870, 11,871, 11,872, 11,875, and 12,000 eV) to create As speciation maps using sparse energy excitation X-ray absorption spectroscopy (SEE-XAS). At locations of interest, at least 3 replicate micro X-ray absorption near edge spectroscopy (μXANES) scans were taken. The incident beam energy was calibrated using sodium arsenate such that the first inflection point was 11,874.0 eV. To investigate the distribution and speciation of S present in the rice grains, some samples were analyzed at beamline 14–3 with an incident beam size of 5 μm and energy of 2.5 keV. The sample, inside of a He-filled chamber, was rastered across the beam at step sizes of 5 μm with a dwell time of 50 ms pixel^−1^. At locations of interest, at least 3 replicate μXANES scans were taken. The incident beam energy was calibrated using sodium thiosulfate peak fluorescence at 2473.05 eV. Samples were also scanned at multiple energies (2472.35, 2473.05, 2473.5, 2474.1, 2476.3, 2480.3, 2482.5, and 2500.0 eV) to create S speciation maps using SEE-XAS. At all beamlines, the sample was placed 45° from the incident beam and a Vortex silicon drift detector was placed 90° from the incident beam.

The SR-μXRF data were processed using the Microprobe Analysis Toolkit (SMAK v 1.66)^70^. Whole-grain images were smoothing using Gaussian blurring and stretched in the horizontal direction to correct for placing the sample at 45°. Speciation maps were constructed by fitting the multiple energy maps to normalized spectra from reference standards. For As, this included arsenite glutathione, sodium arsenite, and DMA. For S, speciation maps were constrained by first fitting the μXANES (described below).

The μXANES data were processed using Athena 0.9.26. After background removal and normalization, samples were fit to known standards using linear combination fitting. For As, standards considered included arsenite glutathione, sodium arsenite, DMA, and sodium arsenate (not detected in μXANES). For S, standard spectra were collected for reduced glutathione (GSH), oxidized glutathione (GSSG), sulfate, methionine sulfoxide (MetO), dimethyl sulfoxide (DMSO), adenosine 5′-phosphosulfate (APS), biotin, *S*-adenosyl methionine (SAM), and thiamine. Most S compounds were analyzed as 0.1 M solutions placed between S-free tape and 3-μm thick Mylar film. SAM and biotin were finely ground and dusted onto S-free tape. Sulfur speciation in the grains were best fit by a combination of GSH, GSSG, DMSO, and APS.

## Supplementary Information

Supplementary Information.

## References

[1] Zhao FJ, McGrath SP, Meharg AA (2010). Arsenic as a food chain contaminant: mechanisms of plant uptake and metabolism and mitigation strategies. Annu. Rev. Plant Biol..

[2] IARC (2012). Arsenic and arsenic compounds monograph. IARC Monogr. Eval. Carcinog. Risks Hum..

[3] Jia Y (2013). Microbial arsenic methylation in soil and rice rhizosphere. Environ. Sci. Technol..

[4] Lomax C (2012). Methylated arsenic species in plants originate from soil microorganisms. New Phytol..

[5] Zhao FJ, Zhu YG, Meharg AA (2013). Methylated arsenic species in rice: geographical variation, origin, and uptake mechanisms. Environ. Sci. Technol..

[6] Hansen HR (2011). Identification of tetramethylarsonium in rice grains with elevated arsenic content. J. Environ. Monit..

[7] Rahman H (2019). Modifying the parboiling of rice to remove inorganic arsenic, while fortifying with calcium. Environ. Sci. Technol..

[8] Tang Z (2020). Dimethylarsinic acid is the causal agent inducing rice straighthead disease. J. Exp. Bot..

[9] Limmer MA, Wise P, Dykes GE, Seyfferth AL (2018). Silicon decreases dimethylarsinic acid concentration in rice grain and mitigates straighthead disorder. Environ. Sci. Technol..

[10] Le XC (2000). Determination of monomethylarsonous acid, a key arsenic methylation intermediate, in human urine. Environ. Health Perspect..

[11] Seyfferth AL, Limmer MA, Dykes GE (2018). On the use of silicon as an agronomic mitigation strategy to decrease arsenic uptake by rice. Adv. Agron..

[12] Raab A, Williams PN, Meharg A, Feldmann J (2007). Uptake and translocation of inorganic and methylated arsenic species by plants. Environ. Chem..

[13] Heitkemper DT, Kubachka KM, Halpin PR, Allen MN, Shockey NV (2009). Survey of total arsenic and arsenic speciation in us-produced rice as a reference point for evaluating change and future trends. Food Addit. Contam. Part B Surveill..

[14] Savant NK, Snyder GH, Datnoff LE (1996). Silicon management and sustainable rice production. Adv. Agron..

[15] Liu WJ, McGrath SP, Zhao FJ (2014). Silicon has opposite effects on the accumulation of inorganic and methylated arsenic species in rice. Plant Soil.

[16] Ma JF (2008). Transporters of arsenite in rice and their role in arsenic accumulation in rice grain. Proc. Natl. Acad. Sci..

[17] Guo W, Zhang J, Teng M, Wang LH (2009). Arsenic uptake is suppressed in a rice mutant defective in silicon uptake. J. Plant Nutr. Soil Sci..

[18] Fleck AT, Mattusch J, Schenk MK (2013). Silicon decreases the arsenic level in rice grain by limiting arsenite transport. J. Plant Nutr. Soil Sci..

[19] Meharg C, Meharg AA (2015). Silicon, the silver bullet for mitigating biotic and abiotic stress, and improving grain quality, in rice?. Environ. Exp. Bot..

[20] Seyfferth AL, Fendorf S (2012). Silicate mineral impacts on the uptake and storage of arsenic and plant nutrients in rice (*Oryza sativa* L.). Environ. Sci. Technol..

[21] Seyfferth AL (2016). Soil incorporation of silica-rich rice husk decreases inorganic arsenic in rice grain. J. Agric. Food Chem..

[22] Luxton TP, Tadanier CJ, Eick MJ (2006). Mobilization of arsenite by competitive interaction with silicic acid. Soil Sci. Soc. Am. J..

[23] Dykes GE, Chari NR, Seyfferth AL (2020). Si-induced DMA desorption is not the driver for enhanced DMA availability after Si addition to flooded soils. Sci. Total Environ..

[24] Smith E (2009). Localization and speciation of arsenic and trace elements in rice tissues. Chemosphere.

[25] Choi SH (2014). Analysis of arsenic in rice grains using ICP-MS and fs LA-ICP-MS. J. Anal. At. Spectrom..

[26] Meharg AA (2008). Speciation and localization of arsenic in white and brown rice grains. Environ. Sci. Technol..

[27] Seyfferth AL, Webb SM, Andrews JC, Fendorf S (2011). Defining the distribution of arsenic species and plant nutrients in rice (*Oryza sativa* L.) from the root to the grain. Geochim. Cosmochim. Acta.

[28] Lombi E (2009). Speciation and distribution of arsenic and localization of nutrients in rice grains. New Phytol..

[29] Zheng MZ (2011). Spatial distribution of arsenic and temporal variation of its concentration in rice. New Phytol..

[30] Jo G, Todorov TI (2019). Distribution of nutrient and toxic elements in brown and polished rice. Food Chem..

[31] Wu TL (2019). Speciation and location of arsenic and antimony in rice samples around antimony mining area. Environ. Pollut..

[32] Krishnan S, Dayanandan P (2003). Structural and histochemical studies on grain-filling in the caryopsis of rice (*Oryza sativa* L.). J. Biosci..

[33] Moore KL, Lombi E, Zhao F-J, Grovenor CRM (2012). Elemental imaging at the nanoscale: nanoSIMS and complementary techniques for element localisation in plants. Anal. Bioanal. Chem..

[34] Carey A-M (2010). Grain unloading of arsenic species in rice. Plant Physiol..

[35] Zheng MZ, Li G, Sun GX, Shim H, Cai C (2013). Differential toxicity and accumulation of inorganic and methylated arsenic in rice. Plant Soil.

[36] Carey A-M (2011). Phloem transport of arsenic species from flag leaf to grain during grain filling. New Phytol..

[37] Punshon T (2018). Elemental distribution in developing rice grains and the effect of flag-leaf arsenate exposure. Environ. Exp. Bot..

[38] Randall PJ, Freney JR, Spencer K (2003). Diagnosing sulfur deficiency in rice by grain analysis. Nutr. Cycl. Agroecosyst..

[39] Blair GJ, Momuat EO, Mamaril CP (1979). Sulfur nutrition of rice. II. Effect of source and rate of s on growth and yield under flooded conditions. Agron. J..

[40] Juliano BO (1987). Effect of soil sulfur deficiency on sulfur amino acids and elements in brown rice. Cereal Chem..

[41] Song W-Y (2014). A rice ABC transporter, OsABCC1, reduces arsenic accumulation in the grain. Proc. Natl. Acad. Sci..

[42] Raab A, Meharg AA, Jaspars M, Genney DR, Feldmann J (2004). Arsenic–glutathione complexes—their stability in solution and during separation by different HPLC modes. J. Anal. At. Spectrom..

[43] Wang J (2020). Thiolated arsenic species observed in rice paddy pore waters. Nat. Geosci..

[44] Ackerman AH (2005). Comparison of a chemical and enzymatic extraction of arsenic from rice and an assessment of the arsenic absorption from contaminated water by cooked rice. Environ. Sci. Technol..

[45] Colina Blanco AE, Kerl CF, Planer-Friedrich B (2021). Detection of thioarsenates in rice grains and rice products. J. Agric. Food Chem..

[46] Fan J, Xia X, Hu Z, Ziadi N, Liu C (2013). Excessive sulfur supply reduces arsenic accumulation in brown rice. Plant, Soil Environ..

[47] Dixit G (2016). Reduced arsenic accumulation in rice (*Oryza sativa* L.) shoot involves sulfur mediated improved thiol metabolism, antioxidant system and altered arsenic transporters. Plant Physiol. Biochem..

[48] Zhang J (2016). Influence of sulfur on transcription of genes involved in arsenic accumulation in rice grains. Plant Mol. Biol. Rep..

[49] Raboy V (2009). Approaches and challenges to engineering seed phytate and total phosphorus. Plant Sci..

[50] Perera I, Seneweera S, Hirotsu N (2018). Manipulating the phytic acid content of rice grain toward improving micronutrient bioavailability. Rice.

[51] Li RY (2009). The rice aquaporin lsi1 mediates uptake of methylated arsenic species. Plant Physiol..

[52] Teasley WA, Limmer MA, Seyfferth AL (2017). How rice (*Oryza sativa* L.) responds to elevated As under different Si-rich soil amendments. Environ. Sci. Technol..

[53] Limmer MA, Mann J, Amaral DC, Vargas R, Seyfferth AL (2018). Silicon-rich amendments in rice paddies: effects on arsenic uptake and biogeochemistry. Sci. Total Environ..

[54] Maher W, Foster S, Krikowa F, Donner E, Lombi E (2013). Measurement of inorganic arsenic species in rice after nitric acid extraction by HPLC-ICPMS: verification using XANES. Environ. Sci. Technol..

[55] Smith PG (2005). X-ray absorption near-edge structure analysis of arsenic species for application to biological environmental samples. Environ. Sci. Technol..

[56] Suess E (2009). Discrimination of thioarsenites and thioarsenates by X-ray absorption spectroscopy. Anal. Chem..

[57] Hagan ND, Upadhyaya N, Tabe LM, Higgins TJV (2003). The redistribution of protein sulfur in transgenic rice expressing a gene for a foreign, sulfur-rich protein. Plant J..

[58] Kuzuhara Y, Isobe A, Awazuhara M, Fujiwara T, Hayashi H (2000). Glutathione levels in phloem sap of rice plants under sulfur deficient conditions. Soil Sci. Plant Nutr..

[59] Oparka KJ, Gates P (1981). Transport of assimilates in the developing caryopsis of rice (*Oryza sativa* L.): ultrastructure of the pericarp vascular bundle and its connections with the aleurone layer. Planta.

[60] Oparka KJ, Gates P (1981). Transport of assimilates in the developing caryopsis of rice (*Oryza sativa* L.): the pathways of water and assimilated carbon. Planta.

[61] Oparka KJ, Gates P (1984). Sink anatomy in relation to solute movement in rice (*Oryza sativa* L.): a summary of findings. Plant Growth Regul..

[62] Yang J, Luo D, Yang B, Frommer WB, Eom J-S (2018). SWEET11 and 15 as key players in seed filling in rice. New Phytol..

[63] Wu X, Liu J, Li D, Liu C-M (2016). Rice caryopsis development I: dynamic changes in different cell layers. J. Integr. Plant Biol..

[64] Wu X, Liu J, Li D, Liu C-M (2016). Rice caryopsis development II: dynamic changes in the endosperm. J. Integr. Plant Biol..

[65] Penido ES, Bennett AJ, Hanson TE, Seyfferth AL (2016). Biogeochemical impacts of silicon-rich rice residue incorporation into flooded soils: implications for rice nutrition and cycling of arsenic. Plant Soil.

[66] Limmer MA, Seyfferth AL (2020). The role of small molecules in restricting rice accumulation of dimethylarsinic acid. Plant Soil.

[67] Linquist BA (2015). Reducing greenhouse gas emissions, water use, and grain arsenic levels in rice systems. Glob. Chang. Biol..

[68] Arao T, Kawasaki A, Baba K, Matsumoto S (2011). Effects of arsenic compound amendment on arsenic speciation in rice grain. Environ. Sci. Technol..

[69] Jackson BP (2015). Fast ion chromatography-ICP-QQQ for arsenic speciation. J. Anal. At. Spectrom..

[70] Webb SM (2011). The MicroAnalysis Toolkit: X-ray fluorescence image processing software. AIP Conf. Proc..

