# Supplementary material for: Altering the localization and toxicity of arsenic in rice grain
Source: Sci Rep. 2022 Mar 25;12:5210. doi: 10.1038/s41598-022-09236-3 (PMC8956569; doi:10.1038/s41598-022-09236-3)
Supplement: Supplementary file 1 — Supplementary Information. [file 41598_2022_9236_MOESM1_ESM.docx]

Supplementary Information:
Altering the Localization and Toxicity of Arsenic in Rice Grain

Matt A. Limmer and Angelia L. Seyfferth*

Department of Plant and Soil Sciences

University of Delaware

Newark, DE 19716

*Corresponding author

Email: angelias@udel.edu

# Additional Figures


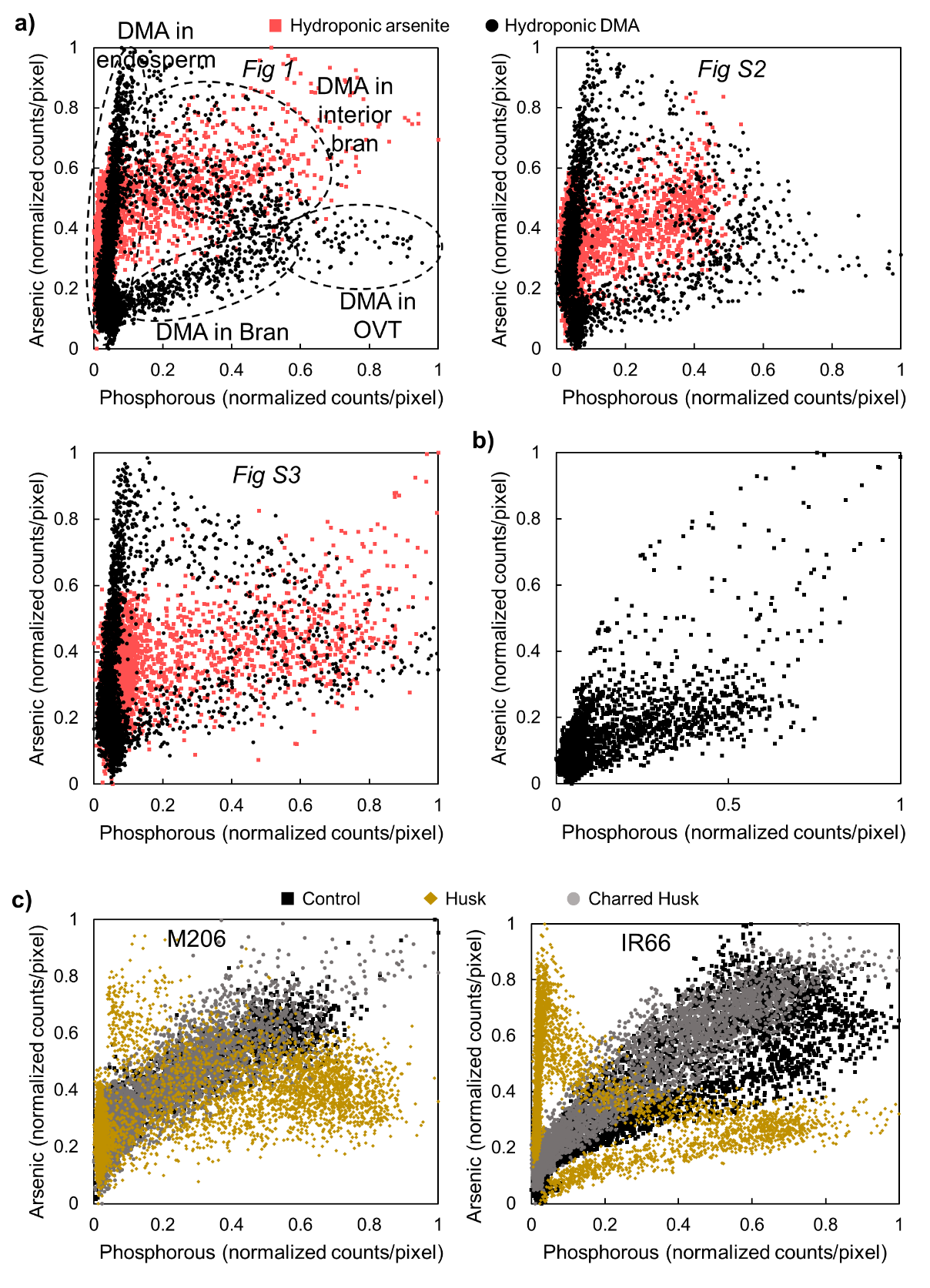


Figure S1 – Grain SR-μXRF As and P correlation plots for a) hydroponic plants (Figures 1, S2, and S3), b) field rice (Figure 5), and c) pot study (Figure 3 and Figure 4). Each point represents a pixel from the corresponding image. Values of P and As were linearly rescaled by the maximum and minimum values. The bran and OVT are characterized by high P, but As intensity in these parts depends on the speciation.


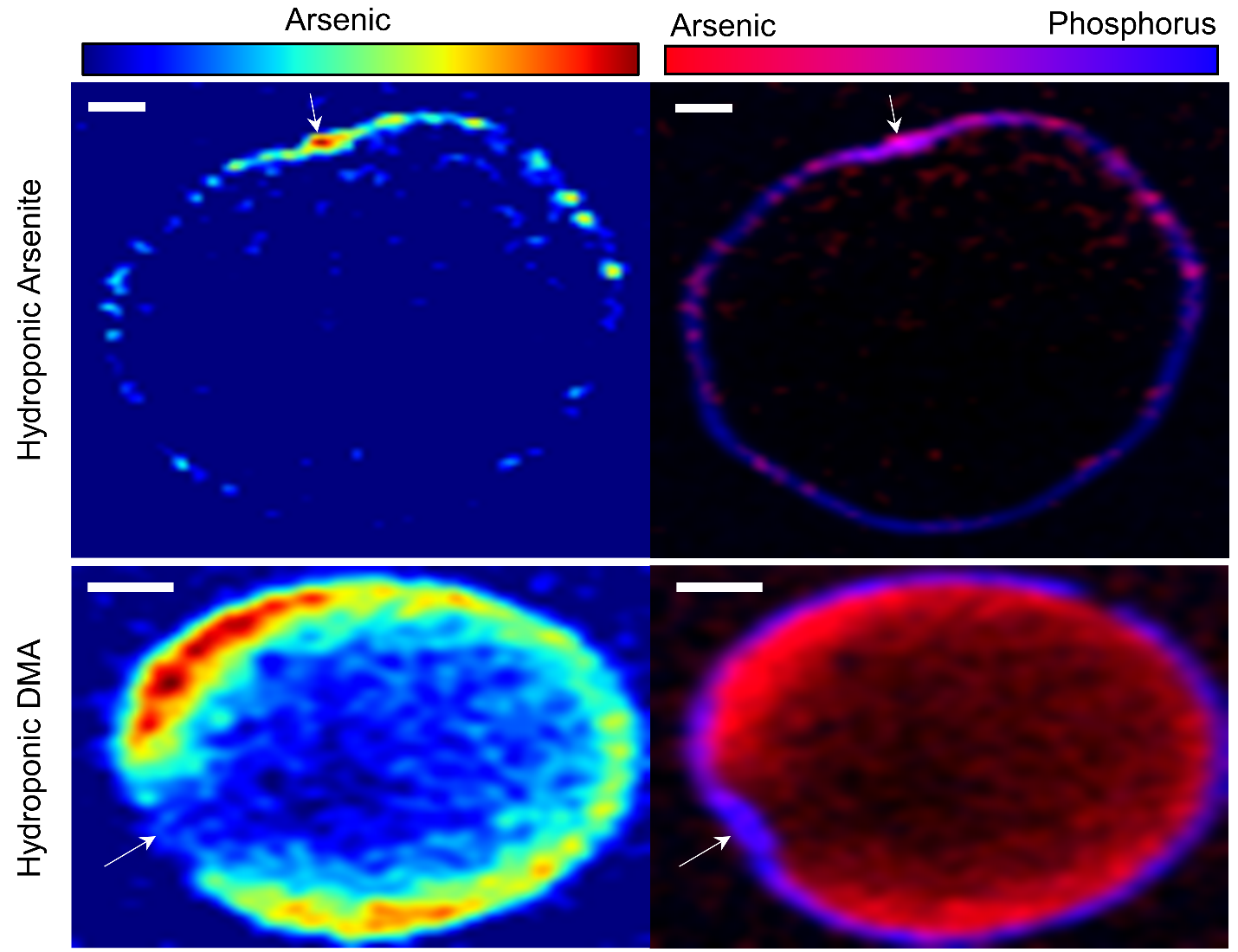


Figure S2 – Distribution of As (left) and colocalization (right) of As (red) and P (blue) in a second set of rice grains grown hydroponically receiving either arsenite (1 uM) or DMA (5 uM). In the grain receiving arsenite, As is mainly located in the bran and the OVT (arrow) as shown by purple hues in the bicolor plots. In the DMA treatment, As permeates into the endosperm and is notably low near the OVT (arrow). Scale bar is 300 μm.


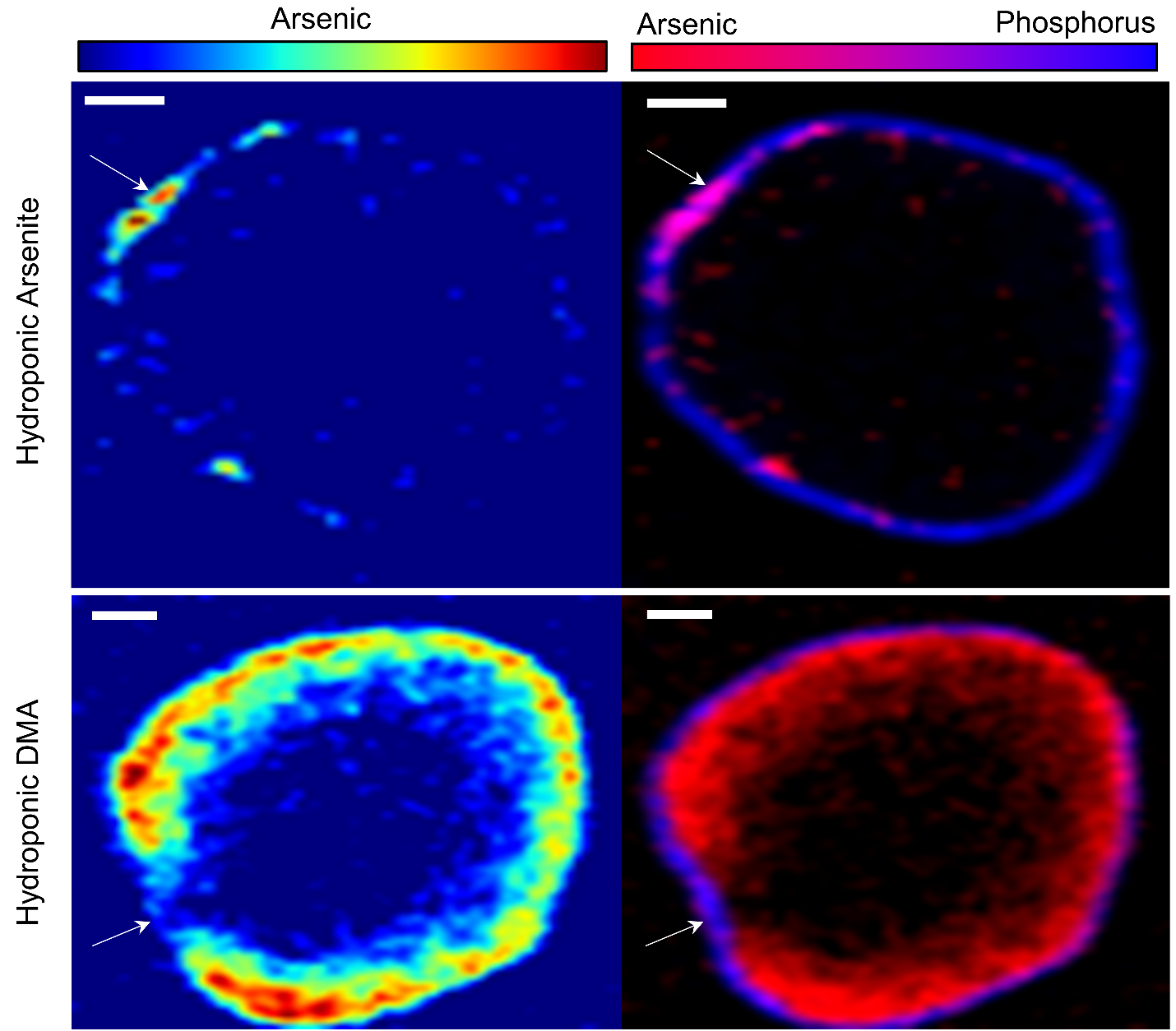


Figure S3 -- Distribution of As (left) and colocalization (right) of As (red) and P (blue) in a third set of rice grains grown hydroponically receiving either arsenite (4 uM) or DMA (5 uM). In the grain receiving arsenite, As is mainly located in the bran and the OVT (arrow) as shown by purple hues in the bicolor plots. In the DMA treatment, As permeates into the endosperm and is notably low near the OVT (arrow). Scale bar is 300 μm.


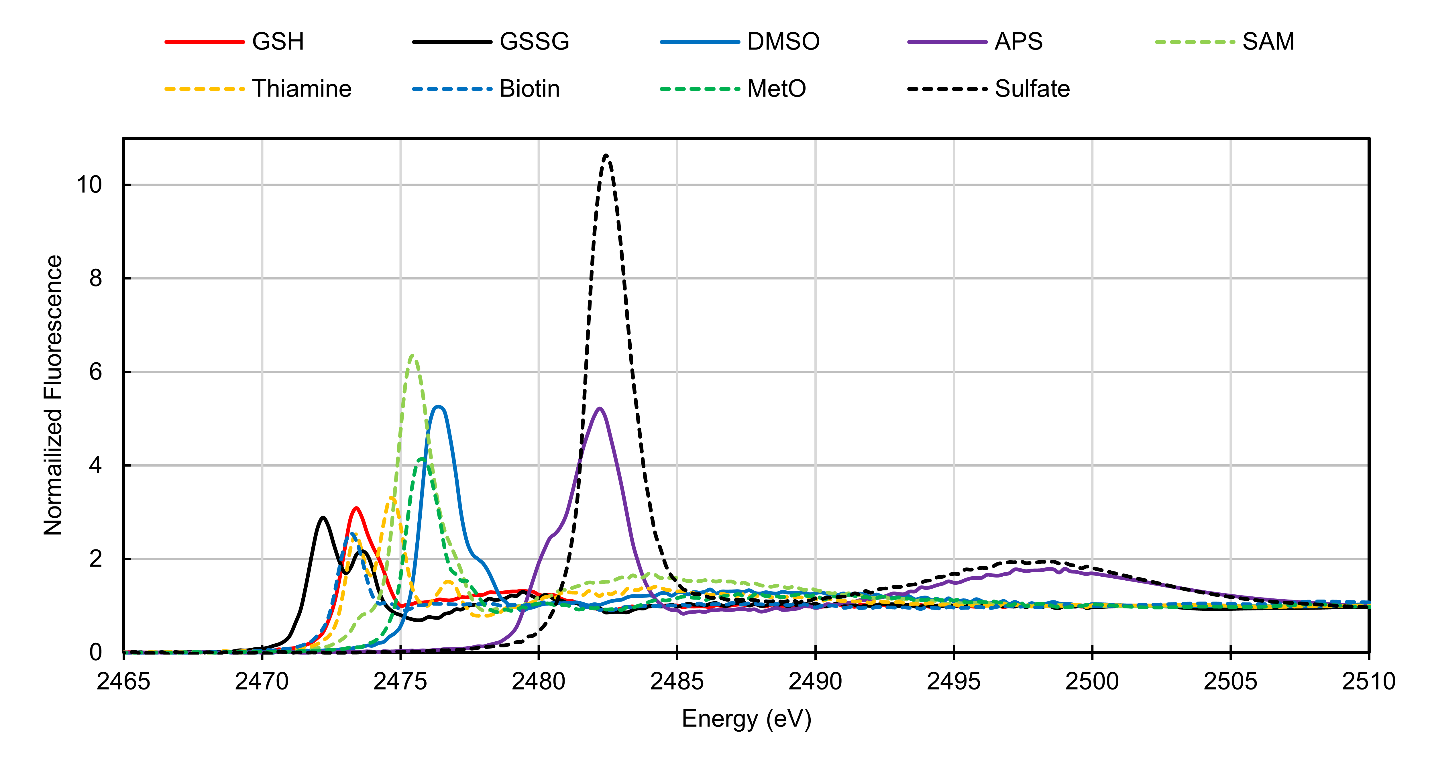


Figure S4 – Sulfur XANES standard spectra. Solid lines indicate standards that were used to fit grain sulfur species.


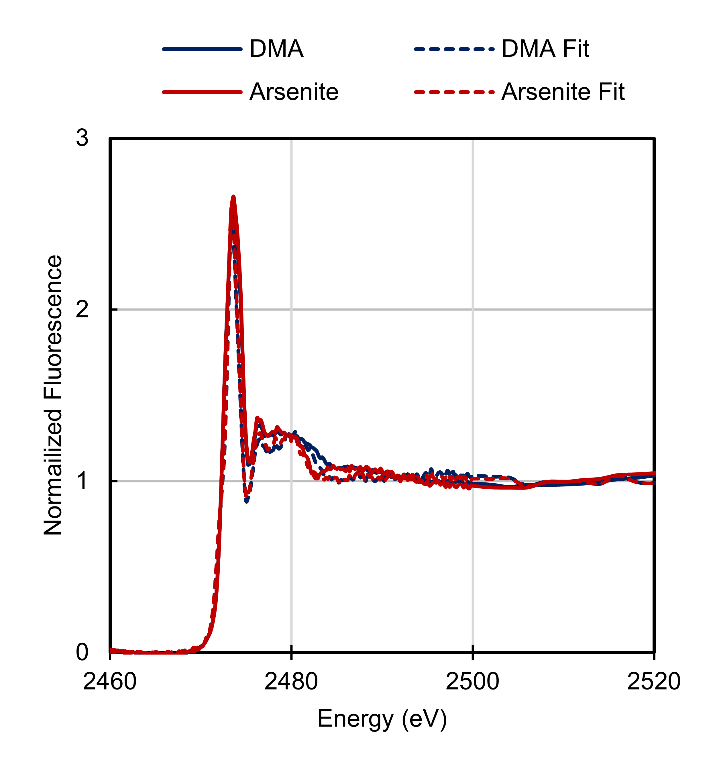


Figure S5 – Sulfur μXANES linear combination fitting from point scans in the bran of hydroponic rice treated with DMA or arsenite.


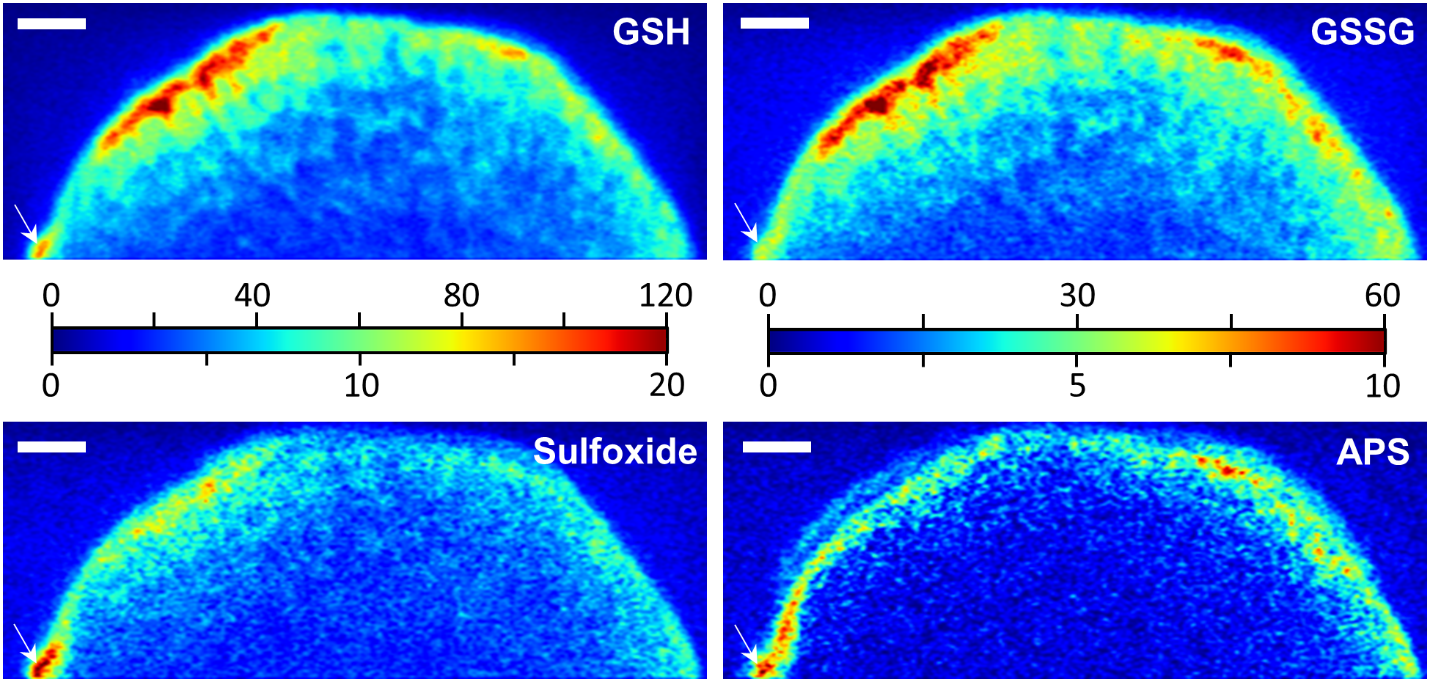


Figure S6 – Distribution of S species in hydroponic rice receiving arsenite. Arrows show the location of the OVT. Each S species is scaled to its maximum value (arbitrary units). Scale bar is 200 μm.


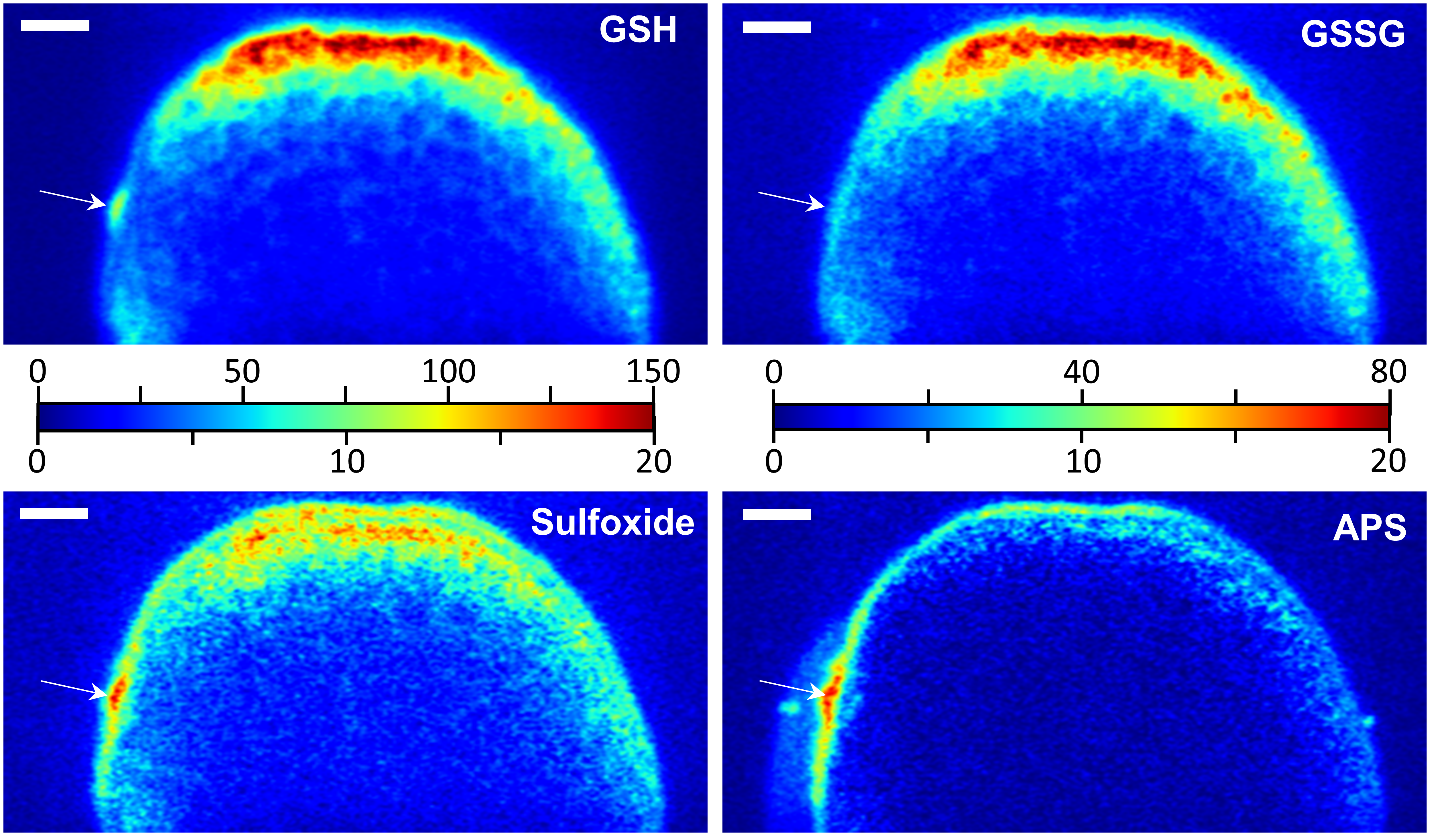


Figure S7 – Distribution of S species in hydroponic rice receiving DMA. Arrows show the location of the OVT. Each S species is scaled to its maximum value (arbitrary units). Scale bar is 200 μm.


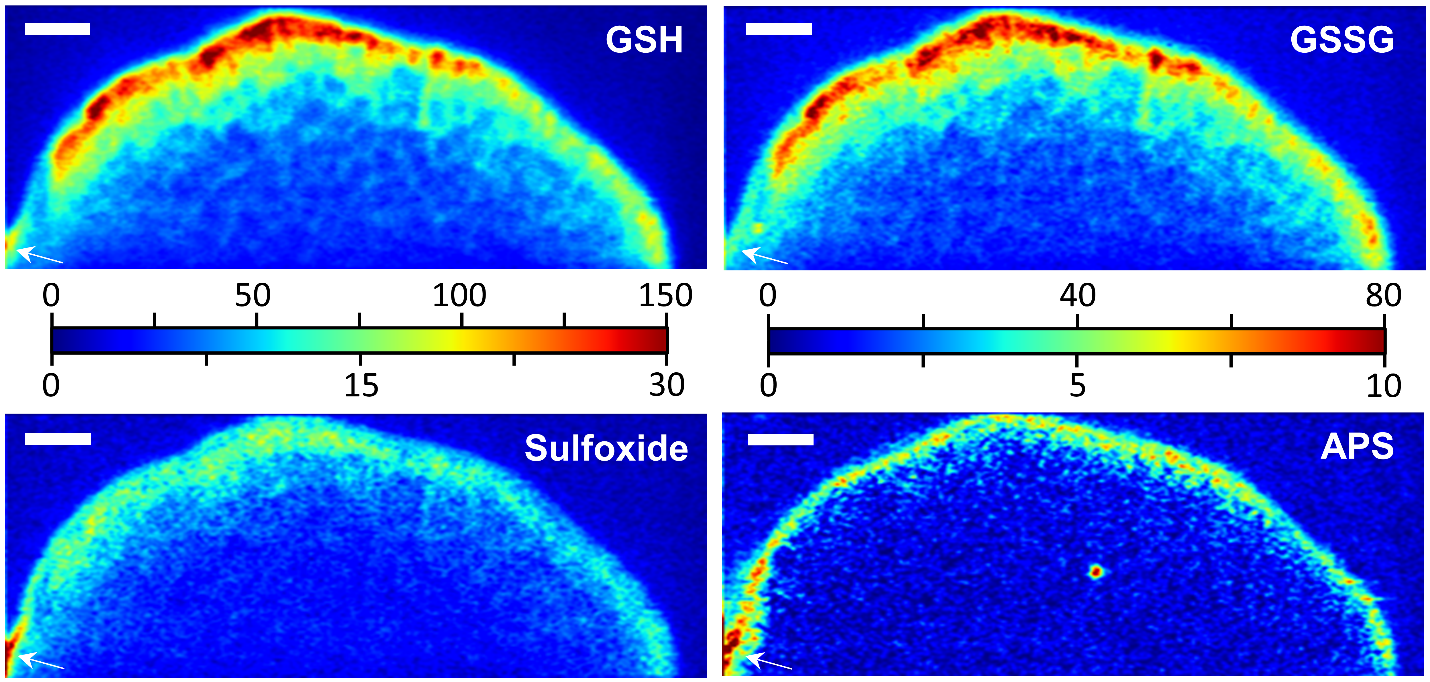


Figure S8 – Distribution of S species in hydroponic rice receiving no arsenic. Arrows show the location of the OVT. Each S species is scaled to its maximum value (arbitrary units). Scale bar is 200 μm.


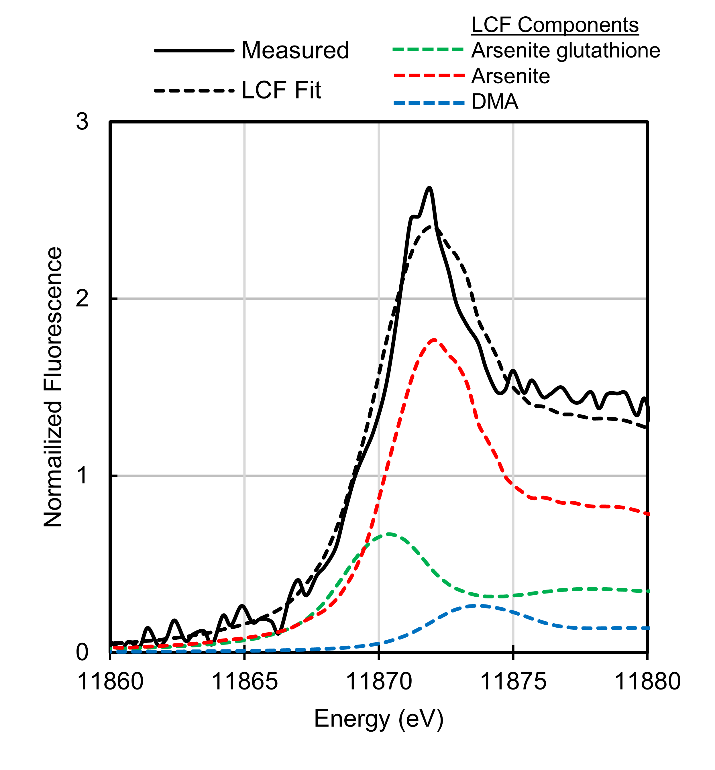


Figure S9 – Linear combination fitting of As μXANES from OVT of field-grown rice (CLXL745). Colored lines indicate the weighted contributions of each As specie.

# Additional Tables

Table S1. Hydroponic nutrient solution composition

| Salt | Concentration (µM) |
| --- | --- |
| Ca(NO_3_)_2_ | 1900 |
| NH_4_NO_3_ | 100 |
| MES | 1000 |
| HNO_3_ | 1000 |
| H_3_PO_4_ | 100 |
| MgSO_4_ | 500 |
| H_3_BO_3_ | 10 |
| Na_2_MoO_4_ | 0.1 |
| ZnCl_2_ | 8 |
| MnCl_2_ | 0.6 |
| CuCl_2_ | 2 |
| NiCl_2_ | 0.1 |
| FeCl_3_ | 20 |
| HEDTA | 57.7 |
| H_4_SiO_4_ | 50 |
| KOH | 100 |
| KCl | 2900 |
| pH adjusted to 6.5 with HCl or NaOH | |

Table S2. Summary of rice grain As concentrations (mg kg^-1^)

| **Experiment** | **Media As** | **Cultivar** | **Treatment** | **Grain As** | **Grain As_i_** | **Grain As_o_** | **Ref** |
| --- | --- | --- | --- | --- | --- | --- | --- |
| Hydroponic | 5 uM | Lemont | DMA | 1.43 | 0.005 | 1.42 | (Limmer & Seyfferth, 2020) |
|  | 1, 4, or 8 uM |  | As(III) | 0.16 | 0.16 | 0.005 |  |
| Pot | 17 mg kg^-1^ | M206 | Control | 0.80 | 0.37 | 0.42 | (Seyfferth *et al.*, 2016) |
|  |  |  | Husk | 0.98 | 0.15 | 0.83 |  |
|  |  |  | Charred husk | 0.64 | 0.29 | 0.36 |  |
|  |  | IR66 | Control | 0.76 | 0.43 | 0.33 |  |
|  |  |  | Husk | 1.13 | 0.20 | 0.93 |  |
|  |  |  | Charred husk | 0.44 | 0.27 | 0.17 |  |
| Field | 4.2 mg kg^-1^ | CLXL745 |  | 0.65 | 0.32 | 0.33 | (Linquist *et al.*, 2015) |

Table S3. Arsenic μXANES linear combination fits of As species in rice bran for IR66. Locations are shown in Fig 4.

| Treatment | Location | r-factor | Arsenite(Glu)_3_ | Arsenite | DMA |
| --- | --- | --- | --- | --- | --- |
| Control | 1 | 0.023 | 29% | 48% | 23% |
|  | 2 | 0.035 | 23% | 65% | 12% |
|  | 3 | 0.020 | 27% | 71% | 2% |
| Husk | 1 | 0.037 | 24% | 28% | 48% |
|  | 2 | 0.022 | 27% | 46% | 26% |
|  | 3 | 0.017 | 33% | 33% | 34% |
| Charred husk | 1 | 0.070 | 6% | 78% | 16% |
|  | 2 | 0.019 | 8% | 81% | 11% |
|  | 3 | 0.016 | 2% | 91% | 6% |
